# Short Sleep Duration Is Positively Associated With Traditional and Non‐Traditional Markers of Adiposity and Cardiometabolic Risk in Individuals at Secondary Healthcare: A Cross‐Sectional Study

**DOI:** 10.1096/fj.202602078RR

**Published:** 2026-08-28

**Authors:** Nathallia Maria Cotta e Oliveira, Mariana De Santis Filgueiras, Gilmara Alves Zanirate, Flávia Galvão Cândido, Sarah Aparecida Vieira Ribeiro, Helen Hermana Miranda Hermsdorff

**Affiliations:** ^1^ Laboratory of Clinical Analysis and Genomics (LACEG); Laboratory of Energy Metabolism and Body Composition (LAMECC), Department of Nutrition and Health Universidade Federal de Viçosa Viçosa Minas Gerais Brazil; ^2^ Department of Nutrition and Health Universidade Federal de Viçosa Viçosa Minas Gerais Brazil; ^3^ Embrapa Alimentos e Territórios Maceió Alagoas Brazil

**Keywords:** abdominal obesity, blood pressure, insulin resistance, overweight, total sleep time

## Abstract

This study aimed to investigate the associations between short and long sleep duration and traditional and non‐traditional markers of adiposity and cardiometabolic risk. This is a cross‐sectional study that enrolled secondary data of 937 men and women ≥ 18 years assisted at Secondary Healthcare. Traditional markers of adiposity and cardiometabolic risk were waist circumference, body mass index, triglycerides, glucose, glycated hemoglobin, systolic (SBP) and diastolic (DBP) blood pressure, while the non‐traditional were deep‐abdominal‐adipose‐tissue index, visceral adiposity index (VAI), lipid accumulation product index, Castelli risk index 1 and 2, TG/HDL‐c ratio, atherogenic index of plasma, and triglyceride‐glucose index (TyG index). Self‐reported habitual sleep durations were classified as short, recommended, or long according to the National Sleep Foundation. The prevalence ratios (PRs) were estimated by Poisson regression models, adjusted for potential confounders (*α* = 0.05). The prevalence of short and long sleep was 9.8% (5.9 ± 0.7 h/night) and 36.7% (10.5 ± 1.3 h/night), respectively. Short sleep was positively associated with abdominal obesity (PR: 1.15; 95% CI: 1.01–1.23), overweight (PR: 1.11; 95% CI: 1.00–1.24), high VAI (PR: 1.36; 95% CI: 1.04–1.77), high TyG index (PR: 1.44; 95% CI: 1.09–1.89), and high DBP (PR: 1.35; 95% CI: 1.07–1.72), regardless of confounders. No association between long sleep and cardiometabolic risk was found. In conclusion, individuals at high cardiovascular risk with short sleep duration are more likely to have adiposity‐related risk, insulin resistance, and diastolic blood pressure imbalance.

## Graphical abstract

This cross‐sectional study involving 937 adults at high cardiovascular risk found a positive association between short sleep duration (< 7 h/night) and traditional and non‐traditional markers of adiposity (abdominal obesity, overweight, and high visceral adiposity index [VAI]), a non‐traditional marker of glucose metabolism disruption (high triglyceride‐glucose [TyG] index), and altered diastolic blood pressure. Longer sleep duration was not associated with cardiometabolic risk. Encouraging patients to sleep at least 7 h/night is an easy and effective strategy for reducing cardiovascular risk.

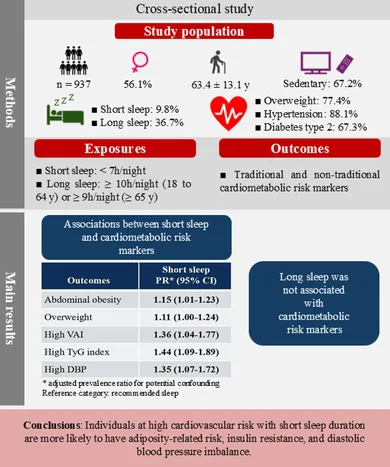

**Abbreviations**
BMI: body mass index
DBP: diastolic blood pressure
HDL‐c: high‐density lipoprotein cholesterol
HOMA‐IR: homeostatic model assessment of insulin resistance
SBP: systolic blood pressure
TG: fasting triglycerides
TyG index: triglyceride‐glucose index
VAI: visceral adiposity index
WC: waist circumference

## 1 Introduction

The cardiometabolic risk markers encompass a set of factors triggered by abnormalities in glucose and lipid metabolism, an increase in central body fat, and an imbalance in blood pressure [1]. Overweight and obesity are one of the most prevalent markers of cardiometabolic risk and are important contributors to the increase of atherogenic lipoproteins, blood glucose, and blood pressure [1]. Globally, in 2022, approximately 2.5 billion adults were overweight, of which 890 million lived with obesity [2]. Several modifiable risk factors have been associated with the increase of body weight and metabolic dysfunction, such as consumption of ultra‐processed foods [3, 4], physical inactivity [5], and disruption of circadian rhythms [6, 7].

The physiological and metabolic activities, as well as the behavior of mammals, are controlled by a complex cyclical system which involves the interaction of “*zeitgebers*” as the light, a central clock—the suprachiasmatic nucleus, and several peripheral clocks. This system lasts approximately 24 h and is known as circadian rhythm or circadian cycle [8, 9]. Although several factors can influence the circadian rhythm, adequate sleep time and appropriate exposure to artificial light are among the main factors that regulate the biological clock [8]. In this sense, changes in sleep duration lead to circadian misalignment, which in turn has been associated with obesity, disruptions in glucose metabolism, and dyslipidemia [6, 7], increasing the risk for cardiovascular events [10].

In adults and older adults, short sleep duration, classified as less than 7 h/night, has been consistently associated with a high incidence of obesity [11] and higher risk of central obesity [12]. However, most of the observational studies that showed that short sleep may be a risk factor for increased adiposity used traditional adiposity indicators, such as body mass index (BMI) and waist circumference (WC) [11, 12, 13]. In turn, the few studies that have evaluated the associations between sleep duration and non‐traditional indicators of adiposity, such as the visceral adiposity index—VAI and lipid accumulation product—LAP, involved asymptomatic adults in relation to metabolic disorders or with different cardiovascular risk profiles [14, 15].

Moreover, short sleep duration has also been identified as a potential risk factor for metabolic syndrome [16] and cardiovascular diseases [17] in Mendelian randomization studies. On the other hand, the relationship of long sleep with adiposity and cardiometabolic risk so far remains more inconsistent. Some studies have shown that sleeping more than recommended (long sleep) may also be a risk factor for metabolic syndrome [18] and Type 2 diabetes [19]. However, a previous systematic review and meta‐analysis of observational studies have found no association between long sleep and obesity in adults and older adults [20, 21].

To address these limitations, this study aims to evaluate the associations between short and long sleep duration and traditional and non‐traditional markers of adiposity and cardiometabolic risk in individuals with multiple cardiovascular risk factors, a high‐risk profile typical of populations receiving specialized healthcare. We hypothesize that both short and long sleep durations are associated with increased cardiometabolic risk in our sample and that non‐traditional markers can further improve risk stratification related to sleep duration in this population.

## 2 Methods

### 2.1 Study Design and Population

**FIGURE 1:**
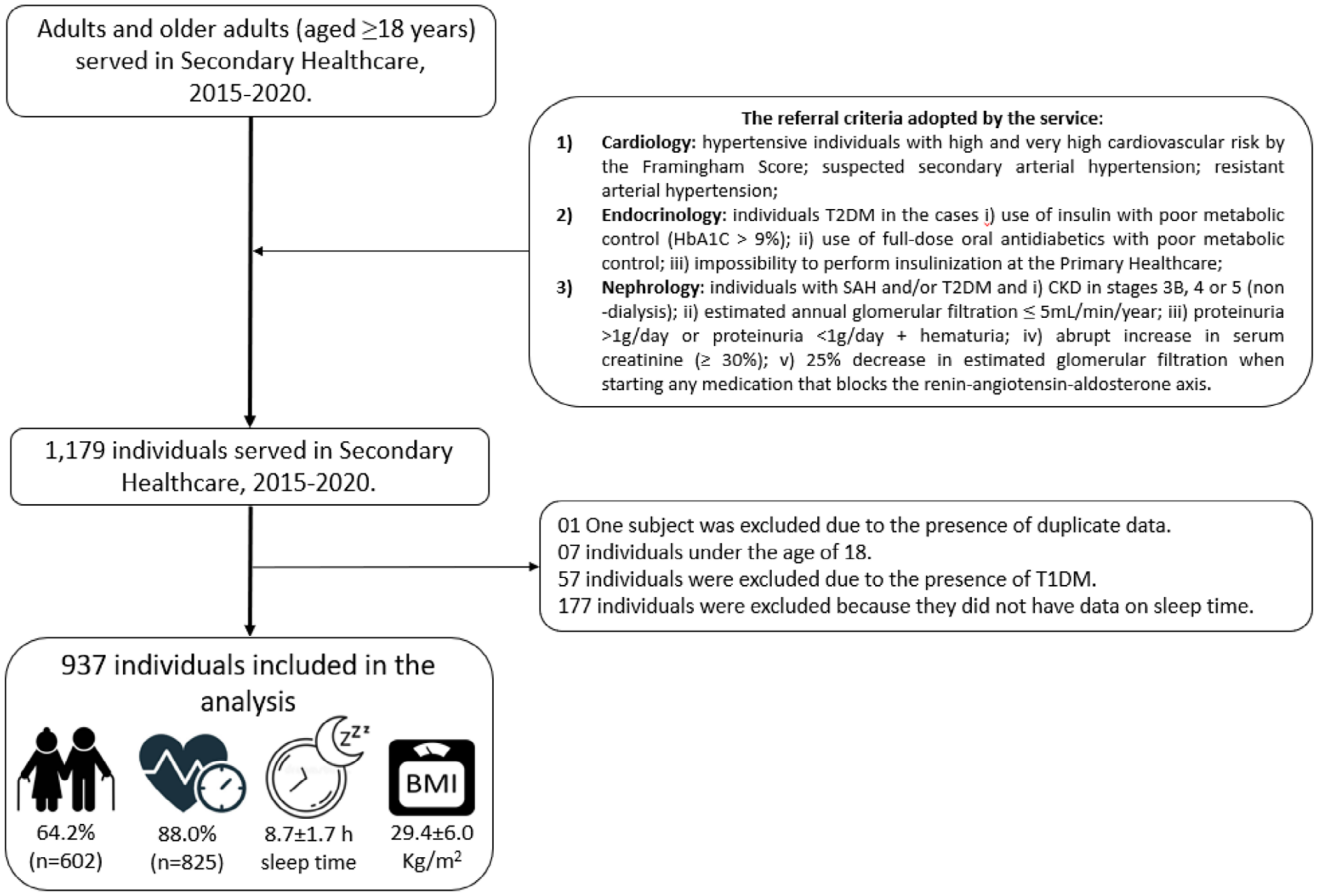
The flowchart depicts the referral criteria utilized by the service and the selection process employed for this study. BMI, body mass index; CKD, chronic kidney disease; HbA1C, glycated hemoglobin; HDL‐c, high‐density lipoprotein cholesterol; SAH, systemic arterial hypertension; T1DM, Type 1 diabetes mellitus; T2DM, Type 2 diabetes mellitus.

The STrengthening the Reporting of OBservational Studies in Epidemiology (STROBE) guideline was used to report the data presented in this article (https://www.strobe‐statement.org/) [22]. This is a cross‐sectional study conducted with baseline data obtained from active medical records of men and women adults and older adults (≥ 18 years) referred for the treatment of noncommunicable chronic diseases in an outpatient Secondary Healthcare center located in Zona da Mata, Minas Gerais, Brazil, from 2015 to 2020. Of the total baseline (*n* = 1179), we exclude individuals with missing values for habitual hours of sleep (*n* = 177), with Type 1 diabetes mellitus (*n* = 57), under 18 years of age (*n* = 7), and with duplicate data (*n* = 1). Thus, the analyses were conducted with 937 individuals (526 women and 411 men). The referral criteria used by the service, according to the area of care [23], and the sample selection process are shown in Figure 1.

The study did not directly involve patients. The managers signed the Institutional Consent Form and the study complied with the provisions of Resolutions 466/12 [24] and 580/18 [25], the latter regarding the ethical peculiarities of studies of strategic interest to the Brazilian Unified Health System. The study was approved by the Ethics Committee of the Universidade Federal de Viçosa (opinion: 5.164.152) and was performed in accordance with the ethical standards as laid down in the 1964 Declaration of Helsinki.

### 2.2 Data Collection

Sociodemographic, lifestyle, and clinical variables were collected from the records of active patients. Sex was classified as “male” and “female”; education as “< 4 years”, “4–8 years”, and “> 8 years”; marital status as “married” and “unmarried/not married (single, widowed, or other)”; self‐perceived health as “excellent, very good, or good” and “regular, poor, or very poor” [26]; and type of residence as “urban” and “rural”. Besides, smoking and alcoholism histories; engagement in physical activity; social vulnerability; polypharmacy; use of insulin; and use of antihypertensive, antidiabetic, and lipid‐lowering drugs were categorized as “yes” and “no”. Food consumption was assessed through a single 24‐h dietary recall (24HR) applied by a trained professional. The 24HR records were registered in the ERICA‐REC24h software. Daily energy intake (kcal/d) was estimated using the Nutritional Composition Tables of Foods Consumed in Brazil [27].

Habitual self‐reported sleep duration (hours/night) was estimated by the interval of questions “what hours do you sleep” and “what hours do you wake up” and classified according to the National Sleep Foundation as follows: (1) *Recommended*: 7 to 9 h of sleep for 18 to 64 years and 7 to 8 h of sleep for ≥ 65 years; (2) *Short*: less than 7 h of sleep for ≥ 18 years; (3) *Long*: 10 or more hours of sleep for 18 to 64 years and 9 or more hours of sleep for ≥ 65 years. In this study, the “may be appropriate” sleep duration category was grouped within the groups of short or long sleep durations [28].

Blood pressure (mmHg), body weight (kg), height (m), and WC (cm) were measured during baseline screening by trained nurses. Blood pressure was measured in the antecubital region using a digital sphygmomanometer (Omron, model HEM 7122) according to standard clinical protocols. Body weight was measured using an electronic scale with a capacity of 300 kg and 100 g increments in lightly clothed subjects. Height was measured using a wall‐mounted vertical stadiometer with a maximum length of 220 cm and an accuracy of 1 mm with an acrylic display, with the subjects barefoot, upright, and with their heels against the wall. WC was measured at the level of the umbilicus scar using an inelastic tape measure.

Blood samples were collected after 10 to 12‐h overnight fasting by venipuncture, and the analyses were performed according to standard protocols in accredited laboratories. The following biochemical variables were collected: fasting glucose (mg/dL), glycated hemoglobin (HbA1C) (%), fasting triglycerides (TG) (mg/dL), total cholesterol (TC) (mg/dL), high‐density lipoprotein cholesterol (HDL‐c) (mg/dL), and low‐density lipoprotein cholesterol (LDL‐c) (mg/dL). TG and HDL‐c were converted into mmol/L using the following conversion factors: TG (dividing mg/dL by 88.57) and HDL‐c (dividing mg/dL by 38.67) [29] for the calculation variables of interest.

### 2.3 Traditional and Non‐Traditional Cardiometabolic Risk Markers

Anthropometric and/or biochemical variables were used to calculate the traditional indicator of adiposity (BMI) [30] and the non‐traditional indicators (deep‐abdominal‐adipose‐tissue index—DAAT, VAI, and lipid accumulation product index—LAP) [31, 32, 33]. Other non‐traditional cardiometabolic risk markers (Castelli risk index 1—CRI‐I, Castelli risk index 2—CRI‐II, TG/HDL‐c ratio, atherogenic index of plasma—AIP, and triglyceride‐glucose index—TyG index) were also calculated (for equations and cut‐off points, see Table 1) [34, 35, 36, 37].

**TABLE 1:** Equations used to calculate traditional and non‐traditional cardiometabolic risk markers and the cut‐off points applied in this study.

Cardiometabolic risk markers	Equations	Cut‐off points
Traditional adiposity indicator		
Body mass index—BMI [30]	Weight (kg)/[height (m)]^2^	Overweight BMI ≥ 25.0 kg/m^2^
Non‐traditional adiposity indicators		
Deep abdominal adipose tissue index—DAAT [31]	Female: −278 + [−0.86 × weight (kg)] + [5.19 × WC (cm)] Male: −382.9 + [1.09 × weight (kg)] + [6.04 × WC (cm)] + [−2.29 × BMI]	High DAAT ≥ 174.17^a^
Visceral adiposity index—VAI [32]	Female: [WC (cm)/(36.58 + 1.89 × BMI (kg/m^2^))] × (triglycerides (mmol/L)/0.81) × (1.52/HDL (mmol/L)) Male: [WC (cm)/(39.69 + 1.88 × BMI (kg/m^2^))] × (triglycerides (mmol/L)/1.03) × (1.31/HDL (mmol/L))	High VAI ≥ 2.49^a^
Lipid accumulation product index—LAP [33]	Female: [(WC (cm)−58) × triglycerides (mmol/L)] Male: [(WC (cm)−65) × triglycerides (mmol/L)]	High LAP ≥ 66.32^a^
Non‐traditional atherogenic indices		
Castelli's risk index I—CRI‐I [34]	TC (mg/dL)/HDL‐c (mg/dL)	High CRI‐I ≥ 4.00^a^
Castelli's risk index II—CRI‐II [34]	LDL (mg/dL)/HDL‐c (mg/dL)	High CRI‐II ≥ 2.19^a^
Triglyceride/high‐density lipoprotein cholesterol ratio—TG/HDL‐c ratio [35]	TG (mg/dL)/HDL‐c (mg/dL)	High TG/HDL‐c ratio ≥ 3.33^a^
Atherogenic Index of Plasma—AIP [36]	log [triglycerides (mg/dL)/HDL (mg/dL)]	High AIP ≥ 1.20^a^
Non‐traditional insulin resistance index		
Triglyceride‐glucose index—TyG index [37]	ln [(fasting triglycerides (mg/dL) × fasting glucose (mg/dL)/2)]	TyG index ≥ 9.24^a^

^a^ The established values correspond to the 50th percentile of the present study sample, due to the absence of well‐established reference thresholds for the studied population.

Besides, other traditional cardiometabolic risk markers were outcomes of the study as abdominal obesity defined by WC ≥ 90 cm and ≥ 80 cm for male and female, respectively [38]; hypertriglyceridemia defined by values of TG ≥ 150 mg/dL; high TC by values ≥ 190 mg/dL; high LDL‐c by values ≥ 130 mg/dL; low HDL‐c by values < 50 mg/dL and < 40 mg/dL for men and women, respectively [39]; high fasting glucose by values ≥ 100 mg/dL; high HbA1c by values ≥ 6.5% [40]; high systolic blood pressure (SBP) by values ≥ 140 mmHg; high diastolic blood pressure (DBP) by values ≥ 90 mmHg [41]; diabetes defined by the presence of at least one of the following variables: fasting glucose ≥ 126 mg/dL, HbA1c ≥ 6.5%, and/or use of anti‐diabetic drugs [40]; hypertension defined by the presence of at least one of the following variables: SBP/DBP ≥ 140/90 mmHg, use of anti‐hypertensive drugs, and/or history of hypertension [41]; and metabolic syndrome determined by the presence of WC ≥ 90 cm for men and ≥ 80 cm for women associated with two or more of the following criteria: (1) SBP/DBP ≥ 130/85 mmHg, use of anti‐hypertensive drugs, and/or history of hypertension; (2) fasting glucose ≥ 100 mg/dL and/or history of Type 2 diabetes; (3) TG ≥ 150 mg/dL and/or use of lipid‐lowering drugs; and (4) low HDL‐c (men < 40 mg/dL and women < 50 mg/dL) and/or use of lipid‐lowering drugs [42].

### 2.4 Statistical Analysis

The statistical analysis was carried out using SPSS version 21.0 (Statistical Package for the Social Sciences, IBM, Chicago, IL) and STATA version 14.0 (Stata Corporation, College Station, Texas, USA). The level of significance in two‐tailed tests was set at 5%.

**FIGURE 2:**
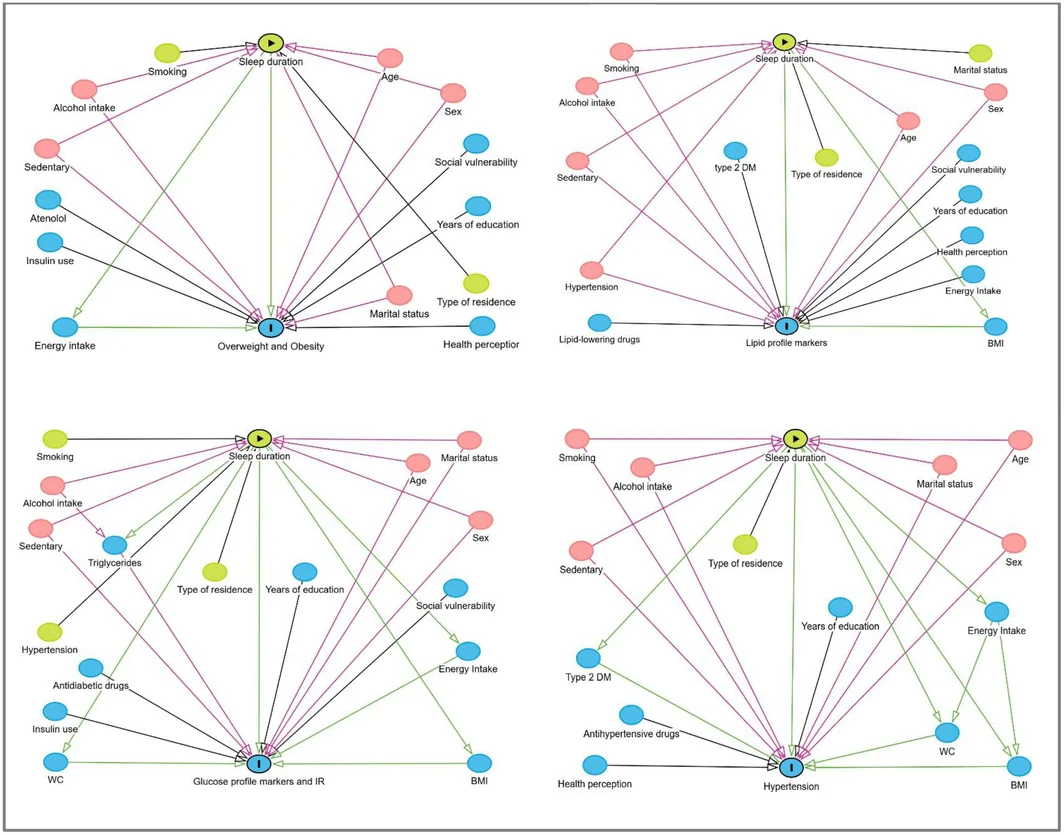
Theoretical model to summarize the relationships between sleep duration (exposure) with outcomes related to adiposity, lipid and glucose profiles, and blood pressure. BMI, body mass index; Type 2 DM, Type 2 diabetes mellitus; WC, waist circumference. By https://www.dagitty.net/ [61].

Categorical variables were described as absolute (*n*) and relative (%) frequencies and compared by Pearson's Chi‐square test. Continuous variables were compared by one‐way ANOVA followed by post hoc Bonferroni test and described as mean and standard deviation (SD). To assess the associations between short and long sleep (independent variable) and traditional and non‐traditional cardiometabolic risk markers (binary dependent variables), Poisson regression models with robust variance estimation [all outcomes with prevalence > 10%] were conducted. Data from regression analysis were presented as prevalence ratio (PR) and their respective 95% confidence intervals (95% CI). The recommended sleep duration was considered the reference category. The multiple models were adjusted by the set of the following criteria:

1. Theoretical model constructed with data obtained from the literature [43, 44, 45, 46, 47, 48, 49, 50, 51, 52, 53, 54, 55, 56, 57, 58, 59, 60]. To identify the minimum fit of the multiple model (confounding variables), directed acyclic graphs (DAGs) were constructed (Figure 2) in the DAGitty program (http://www.dagitty.net/) [61];
2. Statistical criteria (*p*‐value < 0.20 in the bivariate analysis between each independent variable and each dependent variable). Thus, those independent variables (sociodemographic, lifestyle, clinical, and energy intake) that presented a *p*‐value < 0.20 in the association with the dependent variables in the simple Poisson regression models with robust variance estimation were included in the multiple models.

The study's power was calculated based on a two‐sided 95% confidence interval. The effect size was approximately 80%, derived from the differences in sleep duration means (difference = 0.329) and sleep duration SD in the normal and high diastolic blood pressure groups (8.828 ± 1.75 vs. 8.499 ± 1.70), according to the OpenEpi online software (https://www.openepi.com/Menu/OE_Menu.htm) [62].

## 3 Results

Of the total sample (*n* = 937), the mean age was 63.4 ± 13.1 years, and most were women (56.1%), married (59.4%), had few years of education (51.0%), and lived in urban areas (75.5%). Regarding lifestyle, 67.2% did not practice any physical activity, 15.6% were smokers, and 19.4% consumed alcoholic beverages.

The habitual sleep duration was 8.7 (SD 1.8) hours/night. Short and long sleep durations were observed, respectively, in 9.8% and 36.7% of the participants, with mean durations of 5.9 (SD 0.7) and 10.5 (SD 1.3) hours/night, respectively (Table 2).

**TABLE 2:** Characteristics of individuals at high cardiovascular risk according to sleep duration in Secondary Healthcare facility (2015–2020, *n* = 937).

Characteristics	Total	Sleep duration			*p* *
		Short sleep (*n* = 92)	Recommended sleep (*n* = 501)	Long sleep (*n* = 344)	
Sociodemographic and lifestyle					
Sleep duration (h/night)^†^	8.7 (SD 1.8)	5.9 (SD 0.7)^a^	8.0 (SD 0.7)^b^	10.5 (SD 1.3)^c^	< **0.001**
Age (years)^†^	63.4 (SD 13.1)	60.4 (SD 12.1)^a^	60.1 (SD 12.5)^a^	69.1 (SD 12.2)^b^	< **0.001**
Female^‡^	526 (56.1)	52 (56.5)	268 (53.5)	206 (59.9)	0.184
Education^‡^					**0.046**
< 4 years	398 (51.0)	38 (46.3)	206 (47.5)	154 (58.1)	
4–8 years	218 (27.9)	26 (31.7)	124 (28.6)	68 (25.7)	
> 8 years	165 (21.1)	18 (21.9)	104 (23.9)	43 (16.2)	
Married^‡^	542 (59.4)	55 (60.4)	302 (62.1)	185 (55.2)	0.137
Social vulnerability, yes^‡^	95 (12.1)	9 (12.2)	63 (14.9)	23 (8.0)	**0.023**
Type of residence^‡^					0.114
Urban	604 (75.5)	63 (81.8)	330 (76.9)	211 (71.8)	
Rural	196 (24.5)	14 (18.2)	99 (23.1)	83 (28.2)	
Smoking history, yes^‡^	139 (15.6)	16 (17.9)	87 (18.2)	36 (11.1)	**0.019**
Alcoholism history, yes^‡^	173 (19.4)	15 (16.9)	112 (23.5)	46 (14.2)	**0.004**
Sedentary^‡^	597 (67.2)	56 (62.2)	300 (63.4)	241 (73.9)	**0.005**
Clinical					
Health perception, regular/poor/very poor^‡^	411 (51.8)	41 (48.8)	249 (58.0)	121 (43.1)	< **0.001**
Polypharmacy, yes^‡^	623 (70.7)	63 (73.3)	314 (66.7)	246 (75.9)	**0.016**
Use of antihypertensive drugs, yes^‡^	766 (81.8)	73 (79.4)	392 (78.3)	301 (87.5)	**0.002**
Use of antidiabetic drugs, yes^‡^	533 (56.9)	54 (58.7)	297 (59.3)	182 (52.9)	0.172
Use of lipid‐lowering drugs, yes^‡^	523 (73.4)	54 (78.3)	275 (73.5)	194 (71.9)	0.558
Use of insulin, yes^‡^	227 (24.2)	20 (21.7)	129 (25.8)	78 (22.7)	0.498
Daily energy intake (kcal)^†^	1437.1 (SD 708.7)	1365.2 (SD 673.6)	1499.9 (SD 750.8)^a^	1362.9 (SD 643.3)^b^	**0.016**

^†^ One‐way ANOVA with Bonferroni post hoc test. Data are expressed as mean (standard deviation, SD).

^‡^ Pearson's chi‐square test. Data are expressed as absolute, *n* (relative, %) frequencies.

* Bold values indicate statistically significant differences (*p*‐values < 0.05). Different superscript letters indicate statistically significant differences (*p*‐value < 0.05).

The prevalence of hypertension and Type 2 diabetes mellitus was high in the study population (88.1% and 67.3%, respectively), with the metabolic syndrome identified in 35.2% of the sample (Table 3). The high prevalence of Non‐Communicable Disease was accompanied by high use of antihypertensive (81.8%), antidiabetic (56.9%), and lipid‐lowering drugs (73.4%) (Table 2). Moreover, most participants had overweight (77.4%) and abdominal obesity (83.6%) (Table 3).

**TABLE 3:** Traditional and non‐traditional cardiometabolic risk markers of individuals at high cardiovascular risk according to sleep duration in Secondary Healthcare facility (2015–2020, *n* = 937).

Cardiometabolic risk markers	Total	Sleep duration			*p* *
		Short sleep (*n* = 92)	Recommended sleep (*n* = 501)	Long sleep (*n* = 344)	
Abdominal obesity^a^	667 (83.6)	71 (94.7)	354 (82.1)	242 (82.9)	**0.024**
Overweight^a^	700 (77.4)	79 (87.8)	370 (76.5)	251 (76.1)	**0.046**
High DAAT^a^	398 (50.2)	37 (49.3)	213 (49.9)	148 (50.9)	0.956
High VAI^a^	225 (49.9)	28 (65.1)	124 (49.6)	73 (46.2)	0.088
High LAP^a^	241 (50.3)	28 (59.6)	143 (53.6)	70 (42.4)	**0.033**
Hypertriglyceridemia^a^	282 (49.5)	33 (55.9)	164 (52.1)	85 (43.4)	0.093
High total cholesterol^a^	279 (45.7)	28 (45.9)	162 (48.8)	89 (41.0)	0.202
High LDL cholesterol^a^	130 (25.4)	11 (22.5)	80 (28.1)	39 (21.9)	0.295
Low HDL cholesterol^a^	293 (50.3)	30 (52.6)	159 (50.3)	104 (49.5)	0.917
High AIP^a^	273 (51.8)	34 (62.9)	156 (54.4)	83 (44.6)	**0.026**
High Castelli risk index 1^a^	294 (50.4)	34 (59.7)	166 (52.5)	94 (44.8)	0.074
High Castelli risk index 2^a^	256 (50.6)	25 (51.0)	152 (54.1)	79 (44.9)	0.159
High TG/HDL‐c ratio^a^	271 (50.3)	33 (61.1)	156 (52.5)	82 (43.6)	**0.039**
High TyG index^a^	267 (49.9)	38 (66.7)	150 (51.2)	79 (42.7)	**0.005**
Type 2 diabetes mellitus^a^	631 (67.3)	63 (68.5)	349 (69.7)	219 (66.7)	0.183
High fasting glucose^a^	493 (70.9)	54 (73.9)	280 (73.1)	159 (66.5)	0.178
High glycated hemoglobin^a^	459 (82.4)	51 (83.6)	263 (82.9)	145 (81.0)	0.831
Hypertension^a^	825 (88.1)	82 (89.1)	428 (85.4)	315 (91.6)	**0.024**
High SBP^a^	466 (51.2)	49 (55.7)	239 (48.9)	178 (53.1)	0.337
High DBP^a^	330 (36.2)	46 (52.3)	176 (36.1)	108 (32.2)	**0.002**
Metabolic syndrome^a^	330 (35.2)	39 (42.4)	185 (36.9)	106 (30.8)	0.060

Abbreviations: AIP, atherogenic index of plasma; DAAT, deep‐abdominal‐adipose‐tissue; DBP, diastolic blood pressure; HDL‐c, high‐density lipoprotein cholesterol; LAP, lipid accumulation product; LDL‐c, low‐density lipoprotein cholesterol; SBP, systolic blood pressure; TyG index, triglyceride‐glucose index; VAI, visceral adiposity index.

^a^ Pearson's chi‐square test. Data are expressed in absolute, *n* (relative, %) frequencies. Cut‐off points for cardiometabolic risk markers are presented in Table 1 and in the methodology section.

* Bold values indicate statistically significant differences (*p* < 0.05).

In the exploratory analysis, Pearson's chi‐square test indicated significant associations between sleep duration and abdominal obesity, overweight, high TyG index, and elevated diastolic blood pressure. Conversely, no significant associations were observed between sleep duration and traditional dyslipidemia‐related markers (hypertriglyceridemia, high TC and LDL‐c, and low HDL‐c) or non‐traditional markers (High Castelli risk index 1 and 2). Similarly, classic markers of glycemic alteration (high fasting glucose and HbA1c) were also not associated with sleep duration in these initial analyses (Table 3).

In the regression analysis, individuals with short sleep duration had a higher prevalence of abdominal obesity, overweight, and elevated DBP compared to those with recommended sleep duration, regardless of confounding factors. Similarly, significant associations were found between short sleep and non‐traditional markers of cardiometabolic risk. In the multiple regression model, this exposure was significantly associated with a higher prevalence of high VAI (PR: 1.36; 95% CI: 1.04–1.77) and high TyG index (PR: 1.44; 95% CI: 1.09–1.89). Conversely, no statistically significant associations were observed between short sleep duration and traditional or non‐traditional lipid markers (Table 4).

**TABLE 4:** Associations between sleep duration (independent variable) and traditional and non‐traditional cardiometabolic risk markers in individuals at high cardiovascular risk in Secondary Healthcare facility (2015–2020, *n* = 937).

Outcomes	Sleep duration			Sleep duration		
	Short sleep (*n* = 92)	Recommended sleep (*n* = 501)	Long sleep (*n* = 344)	Short sleep (*n* = 92)	Recommended sleep (*n* = 501)	Long sleep (*n* = 344)
	Model 1 PR (95% CI)			Model 2 PR (95% CI)		
Abdominal obesity†	**1.15 (1.08–1.24)**	1 (Ref.)	1.01 (0.94–1.08)	**1.15 (1.01–1.23)**	1 (Ref.)	0.97 (0.90–1.04)
Overweight†	**1.15 (1.05–1.26)**		0.99 (0.92–1.08)	**1.11 (1.00–1.24)**		1.01 (0.92–1.09)
High DAAT†	0.99 (0.77–1.27)		1.01 (0.87–1.18)	0.92 (0.69–1.21)		0.99 (0.83–1.17)
High VAI†	**1.31 (1.02–1.69)**		0.93 (0.76–1.15)	**1.36 (1.04–1.77)**		1.02 (0.80–1.29)
High LAP†	1.11 (0.86–1.44)		**0.79 (0.64–0.98)**	1.14 (0.87–1.50)		0.89 (0.71–1.12)
Hypertriglyceridemia‡	1.07 (0.84–1.38)		0.83 (0.69–1.01)	1.01 (0.75–1.37)		0.94 (0.76–1.17)
High AIP‡	1.16 (0.92–1.46)		**0.82 (0.68–0.99)**	1.11 (0.84–1.45)		0.89 (0.71–1.12)
High Castelli risk index 1‡	1.14 (0.89–1.44)		0.85 (0.71–1.02)	1.04 (0.75–1.43)		0.96 (0.76–1.21)
High Castelli risk index 2‡	0.94 (0.70–1.27)		0.83 (0.68–1.01)	0.95 (0.67–1.36)		0.97 (0.76–1.25)
High TG/HDL‐c ratio‡	1.16 (0.92–1.48)		0.83 (0.68–1.01)	1.08 (0.81–1.43)		0.88 (0.69–1.12)
High TyG index§	**1.30 (1.05–1.61)**		0.83 (0.68–1.02)	**1.44 (1.09–1.89)**		0.95 (0.75–1.20)
High fasting glucose§	1.01 (0.87–1.17)		0.91 (0.82–1.01)	0.96 (0.81–1.13)		1.03 (0.91–1.16)
High glycated hemoglobin§	1.01 (0.89–1.14)		0.98 (0.89–1.06)	1.09 (0.95–1.25)		1.05 (0.95–1.16)
High SBP¶	1.14 (0.92–1.39)		1.08 (0.95–1.24)	1.09 (0.86–1.38)		1.06 (0.90–1.24)
High DBP¶	**1.45 (1.15–1.83)**		0.89 (0.74–1.09)	**1.35 (1.07–1.72)**		0.94 (0.75–1.18)

*Note:* Data is presented as prevalence ratio (95% confidence interval) according to Poisson regression with robust variance. Recommended sleep: Reference category. Bold values indicate statistical significance. Model 1: Crude. Model 2†: Adjusted by age (years), sex (female or male), education (< 4 years, 4–8 years, > 8 years), marital status (not married or married), smoking history (yes or no), alcoholism history (yes or no), and physical activity (yes or no). Model 2‡: Adjusted by age (years), sex (female or male), marital status (not married or married), social vulnerability (yes or no), smoking history (yes or no), alcoholism history (yes or no), physical activity (yes or no), hypertension (yes or no), antihypertensive drugs (yes or no), antidiabetic drugs (yes or no), and lipid‐lowering drugs (yes or no). Model 2§: Adjusted by age (years), sex (female or male), marital status (not married or married), education (< 4 years, 4–8 years, > 8 years), smoking history (yes or no), alcoholism history (yes or no), physical activity (yes or no), antihypertensive drugs (yes or no), antidiabetic drugs (yes or no), lipid‐lowering drugs (yes or no), use of insulin (yes or no), and health perception (excellent, very good, or good and regular, poor or very poor), Model 2¶: Adjusted by age (years), sex (female or male), education (< 4 years, 4–8 years, > 8 years), marital status (not married or married), smoking history (yes or no), alcoholism history (yes or no), physical activity (yes or no), and antihypertensive drugs (yes or no).

Abbreviations: AIP, atherogenic index of plasma; DAAT, deep‐abdominal‐adipose‐tissue; DBP, diastolic blood pressure. LAP, lipid accumulation product; SBP, systolic blood pressure; TG/HDL‐c ratio, triglyceride to high‐density lipoprotein cholesterol ratio; TyG index, triglyceride‐glucose (TyG) index; VAI, visceral adiposity index.

Regarding long sleep duration, in the unadjusted model, individuals with long sleep duration had a lower prevalence of high LAP and high AIP compared to those with recommended sleep. However, after adjustment for potential confounders, these associations did not remain significant. Furthermore, no other traditional or non‐traditional cardiometabolic risk marker assessed in the study was significantly associated with long sleep duration (Table 4).

## 4 Discussion

**FIGURE 3:**
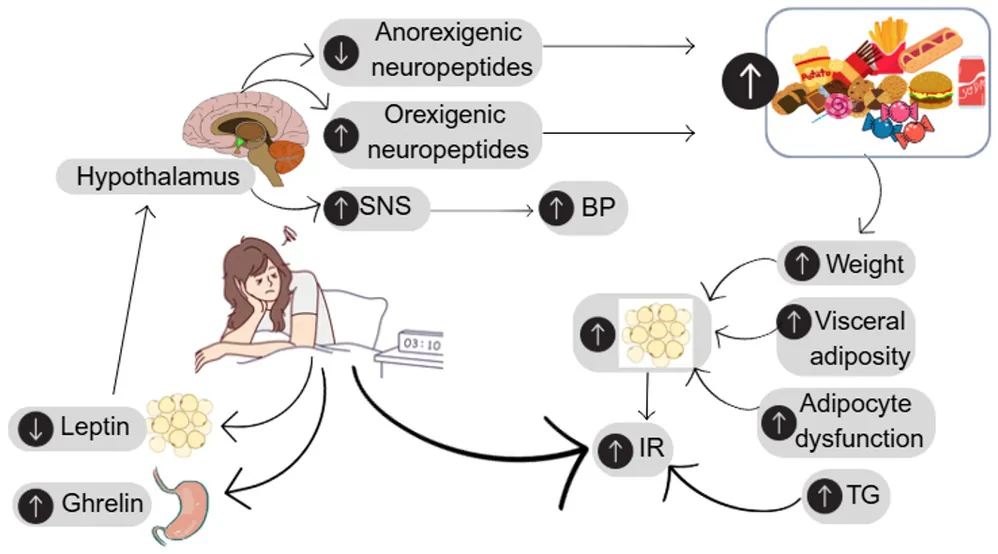
Summaries of the potential mechanisms underlying the relationship between short sleep duration, adiposity, and metabolic disruption. Sleep restriction can alter hypothalamic appetite regulation by upregulating orexigenic and downregulating anorexigenic signaling, thereby driving higher energy intake, total and visceral adiposity, adipose tissue dysfunction, and metabolic disruption. Furthermore, insufficient sleep impairs the insulin signaling cascade and appears to elevate sympathetic nervous system activity, resulting in increased blood pressure. These pathophysiological alterations can significantly increase the risk of cardiovascular diseases. BP, blood pressure; IR, insulin resistance; SNS, sympathetic nervous system; TG, triglycerides.

In this cross‐sectional analysis of adults and older adults at high cardiovascular risk, short habitual sleep duration, classified as less than 7 h/night, was positively associated with adiposity indicators and with worse cardiometabolic risk profiles, the latter represented by disruptions in glucose metabolism and in blood pressure. Results were consistent, and analysis included non‐traditional markers of adiposity and cardiometabolic risk. However, no significant association was found between long habitual sleep duration and the cardiometabolic risk markers. The potential mechanisms involving short sleep duration, adiposity, and metabolic disruption are summarized in Figure 3.

Since the end of the 20th century, studies have shown evidence of the deleterious effects of sleep deprivation for human health, including the increased risk for obesity‐related cardiometabolic disorders [63, 64]. The best described and currently accepted underlying mechanisms of the relationship between short sleep duration and increased risk of obesity, insulin resistance, and Type 2 diabetes involve the imbalance in the concentrations of hormones and/or neuropeptides that act on hunger, satiety, and insulin signaling pathways [15, 64]. Notably, Taheri et al. (2004) [65] showed, in a study assessing sleep by polysomnography enrolling 1024 healthy adults, that reducing average nightly sleep from 8 to 5 h significantly increased ghrelin and reduced leptin, demonstrating a potential underlying mechanism for the associations between short sleep duration and the development of excessive body weight found in our study and in previous longitudinal studies [11, 12, 66, 67, 68].

Leptin and ghrelin are two hormones with antagonistic action that are recognized as the most important regulators of energy balance. Leptin is a hormone synthesized by adipocytes that exerts its action in the hypothalamus through stimulation of the release of anorexigenic neuropeptides that signal the inhibition of feeding and the increase of energy expenditure. Conversely, ghrelin is a gastrointestinal peptide that activates brain pathways responsible for the release of orexigenic neuropeptides, stimulating appetite and food intake [69]. Considering the biological function of these hormones, it is suggested that alterations in their concentrations evidenced during sleep deprivation lead to reduced satiety, increased hunger, and energy intake, and consequently, weight gain and metabolic imbalance [15, 64].

Indeed, it has been well described that hormone production, metabolic processes, as well as food intake in mammals follow a chronobiological rhythm, which repeats approximately every 24 h. Robust evidence has shown that external stressors, such as alterations to the light–dark cycle leading to greater exposure to nighttime light, can cause misalignment of this rhythmic cycle, resulting in negative consequences for normal biological functions and cardiometabolic health [70].

The results from previous epidemiological studies involving populations with diverse cardiovascular risk profiles or healthy individuals have supported the positive associations between short sleep duration and generalist adiposity indices, such as BMI and WC [66, 67, 68], corroborating our results. Interestingly, our study is among the few to investigate the associations between sleep duration and non‐traditional cardiometabolic risk markers linked to central obesity. We found that individuals with short sleep duration (< 7 h/night) had 36% highest prevalence of high VAI compared to those with recommended sleep duration, a result consistent with findings reported by Liu et al. (2024) [15]. Considering the limitations of BMI in distinguishing fat and lean mass, and its inability to identify central fat distribution [71], studies that include alternative markers of central adiposity may offer more robust methodological approaches, reflecting not only adiposity but also the metabolic and endocrine changes of adipose tissue. The VAI is a simple and reliable indicator that assesses concomitantly the central fat distribution and adipocyte metabolic dysfunction, capturing risk parameters unrepresented by traditional markers of adiposity, including increased lipolysis and elevated concentrations of non‐esterified free fatty acids in the portal circulation. Therefore, the VAI provides a more accurate reflection of cardiovascular risk than anthropometric indicators alone [32]. Central fat deposition leads to visceral adipocyte dysfunction, promoting low‐grade chronic inflammation and disruptions in lipid and glucose metabolism [72, 73], which may exacerbate the direct deleterious effects of sleep deprivation on cardiometabolic health.

In our study, short sleep duration was also associated with insulin resistance, defined by the TyG index ≥ 9.24. In the absence of the gold standard method for measuring insulin resistance (i.e., hyperinsulinemic‐euglycemic clamp), epidemiological studies have used surrogate markers such as homeostatic model assessment of insulin resistance (HOMA‐IR) [74] or TyG index [37], both of which show good agreement with the gold standard method. The TyG index has a biological basis linked to insulin resistance, as serum triglyceride accumulation impairs insulin sensitivity in insulin‐dependent cells [37]. Although this index is a reliable indicator comparable to HOMA‐IR for identifying insulin resistance [37], in the Brazilian adult population, the TyG index demonstrated slightly better performance in identifying this clinical condition compared to HOMA‐IR [75]. Furthermore, in other populations, the TyG index has proven to be equal or superior to HOMA‐IR for predicting the risk of cardiovascular events [76, 77].

Our findings were consistent with previous studies demonstrating that sleep restriction impairs glucose metabolism. Observational studies have shown a positive association between insufficient nocturnal sleep duration and insulin resistance [78, 79], a critical pathophysiological precursor to Type 2 diabetes [80]. Likewise, experimental studies have shown that sleep restriction elevates HOMA‐IR in pre‐ and postmenopausal women [81] and reduces insulin sensitivity in healthy men [82].

We also found a positive association between short sleep duration and DBP, although no such association was observed with SBP. However, sleep durations of less than 6 h/night have been associated with increases of 1.73 mmHg in SBP and 2.17 mmHg in DBP [83]. Thereby, previous studies have associated short sleep duration with the development of hypertension [84, 85]. Although mechanisms are not fully understood, increased pro‐inflammatory cytokines, heightened sympathetic nervous system activity, vascular dysfunction, and oxidative stress due to circadian disruption are likely contributors to this association [84, 85].

Our study has limitations. First, habitual sleep duration was self‐reported and was the only available sleep‐related variable. Although self‐reporting may be prone to bias, it is a very reproducible method commonly used in clinical practice, which enhances the applicability of our findings. Nonetheless, including other sleep variables, such as sleep quality, could have provided a more comprehensive understanding of the associations observed. Also, body composition measurements were not available in the medical records. Although this reflects typical outpatient care in the Brazilian public health system, such data could have further reinforced the relationship between short sleep duration and adiposity.

Our study also has several strengths. We evaluated the relationship between sleep duration and non‐traditional cardiometabolic risk markers. Non‐traditional cardiometabolic risk markers offer advantages because they consist of mathematical models capable of reflecting metabolic disruptions, which may remain hidden in the assessment of isolated traditional markers. Thus, by synergistically integrating anthropometric and/or biochemical variables, non‐traditional markers provide better accuracy in cardiovascular risk stratification, ensuring high clinical relevance [32, 86]. Furthermore, we included a representative number of participants; our sample consisted of individuals with multiple cardiometabolic risk factors; data collection procedures were rigorous; data were validated for consistency and reliability; and the associations between sleep duration and cardiometabolic markers were adjusted for a broad range of socioeconomic and lifestyle factors, strengthening the validity of our findings.

## 5 Conclusions

In conclusion, a short sleep duration is positively associated with traditional and non‐traditional markers of adiposity and cardiometabolic risk in individuals at high cardiovascular risk from Secondary Healthcare. These findings reinforce the importance of counseling for a sufficient night's sleep in clinical practice for the prevention and management of overweight, glucose metabolism‐related disorders, and imbalance in blood pressure. Prospective studies are needed to better evaluate these relationships in adults and older adults at high cardiovascular risk.

## Author Contributions

Conceptualization: Nathallia Maria Cotta e Oliveira, Gilmara Alves Zanirate, and Helen Hermana Miranda Hermsdorff. Data curation: Nathallia Maria Cotta e Oliveira, Flávia Galvão Cândido, and Helen Hermana Miranda Hermsdorff. Formal analysis: Nathallia Maria Cotta e Oliveira, Gilmara Alves Zanirate, Mariana De Santis Filgueiras, Sarah Aparecida Vieira Ribeiro, and Helen Hermana Miranda Hermsdorff. Investigation: Nathallia Maria Cotta e Oliveira, Flávia Galvão Cândido, and Helen Hermana Miranda Hermsdorff. Methodology: Nathallia Maria Cotta e Oliveira, Gilmara Alves Zanirate, Flávia Galvão Cândido, and Helen Hermana Miranda Hermsdorff. Project administration, funding acquisition, resources, and supervision: Helen Hermana Miranda Hermsdorff. Writing – original draft: Nathallia Maria Cotta e Oliveira, Gilmara Alves Zanirate, and Helen Hermana Miranda Hermsdorff. Writing – review and editing: Nathallia Maria Cotta e Oliveira, Mariana De Santis Filgueiras, Gilmara Alves Zanirate, Flávia Galvão Cândido, Sarah Aparecida Vieira Ribeiro, and Helen Hermana Miranda Hermsdorff. All authors read and approved the final version of the article.

## Funding

This work was supported by the Coordination for the Improvement of Higher Education Personnel (CAPES) Foundation [Ministry of Education, Brazil, code 001] and HHM Hermsdorff is Research Productivity Fellowship of the National Council for Scientific and Technological Development. CAPES Foundation and CNPq had no role in the study design; in the collection, analysis, and interpretation of data; writing; and the decision to submit the article for publication.

## Conflicts of Interest

The authors declare no conflicts of interest.

## Data Availability

The data that support the findings of this study are available on request from the corresponding author.
